# Factors associated with the use of antihypertensives among seniors

**DOI:** 10.1590/S1518-8787.2016050006458

**Published:** 2016-11-24

**Authors:** Kaio Henrique Correa Massa, José Leopoldo Ferreira Antunes, Maria Lúcia Lebrão, Yeda Aparecida Oliveira Duarte, Alexandre Dias Porto Chiavegatto

**Affiliations:** I Programa de Pós-Graduação em Saúde Pública. Faculdade de Saúde Pública. Universidade de São Paulo. São Paulo, SP, Brasil; II Departamento de Epidemiologia. Faculdade de Saúde Pública. Universidade de São Paulo. São Paulo, SP, Brasil; III Escola de Enfermagem. Universidade de São Paulo. São Paulo, SP, Brasil

**Keywords:** Aged, Drug Utilization, Antihypertensive Agents, Hypertension, prevention & control, Socioeconomic Factors, Health Surveys

## Abstract

**OBJECTIVE:**

Analyze the use of antihypertensives among seniors and the association with socioeconomic and behavioral characteristics.

**METHODS:**

In this seriate cross-sectional study, we used data from the *Saúde, Bem Estar e Envelhecimento* study (SABE – Health, Well-being, and Aging), conducted in 2000, 2006, and 2010 in the city of São Paulo. Association between the use of antihypertensives and the demographic, behavioral, and socioeconomic characteristics and risk factors was analyzed by using multilevel logistic regression models.

**RESULTS:**

We observed increased proportion of use of antihypertensive, from 48.7% in 2000 to 61.3% in 2006, reaching 65.7% in 2010. Among the seniors who made use of this type of medicine, we also observed increased adoption of combined therapy in the period, from 69.9% to 82.6% from 2000 to 2006 and reaching 91.6% in 2010. Multilevel analysis indicated statistically significant increase in use of antihypertensives, even after control by socioeconomic and behavioral characteristics, both in 2006 and in 2010 (OR = 1.90; 95%CI 1.60–2.24 and OR = 1.94; 95%CI 1.62–2.33, respectively). Use of antihypertensives showed positive association with females, higher age group, black skin color, overweight, and smoking history.

**CONCLUSIONS:**

High use of antihypertensives and its association with sociodemographic and behavioral characteristics can help guide the discussion of strategies to improve the epidemiological situation, the quality of life, and the distribution of medicines to the elderly population.

## RESUMO

**OBJETIVO:**

Analisar o uso de medicamentos anti-hipertensivos em idosos e a associação com características socioeconômicas e comportamentais.

**MÉTODOS:**

Neste estudo transversal seriado, foram utilizados dados do estudo SABE (Saúde, Bem Estar e Envelhecimento), realizado em 2000, 2006 e 2010 no município de São Paulo. A associação entre o uso de medicamentos anti-hipertensivos e as características demográficas, socioeconômicas comportamentais e fatores de risco foi analisada por meio de modelos de regressão logísticos multinível.

**RESULTADOS:**

Foi observado aumento da proporção do uso de anti-hipertensivo, de 48,7% em 2000 para 61,3% em 2006, chegando à 65,7% em 2010. Entre os idosos que faziam uso desse tipo de medicamento, também foi observado aumento da adoção da terapia combinada no período, passando de 69,9% para 82,6% de 2000 a 2006 e alcançando 91,6% em 2010. A análise multinível indicou aumento estatisticamente significativo do uso de anti-hipertensivos, mesmo após controle pelas características socioeconômicas e comportamentais, tanto em 2006 como em 2010 (OR = 1,90; IC95% 1,60–2,24 e OR = 1,94; IC95% 1,62–2,33, respectivamente). O uso de anti-hipertensivos apresentou associação positiva com sexo feminino, maior faixa etária, cor da pele preta, sobrepeso e histórico de tabagismo.

**CONCLUSÕES:**

O uso elevado de anti-hipertensivos e sua associação com as características sociodemográficas e comportamentais podem ajudar a orientar a discussão de estratégias para a melhoria do quadro epidemiológico, da qualidade de vida, e da distribuição de medicamentos para a população idosa.

## INTRODUCTION

Systemic Arterial Hypertension (SAH) is considered one of the most important public health problems^26^. In addition to presenting high prevalence and low control rate, it is one of the main risk factors for brain and cardiovascular diseases, recognized as the leading causes of mortality in the world^24^ ^,^ ^26^. It is estimated that two-thirds of cerebrovascular accidents and half of all the burden of coronary heart disease can be attributed to unsatisfactory levels of arterial blood pressure^14^.

SAH is generally classified as primary or secondary. Primary hypertension emerges mainly in middle age and onset of old age, and in the literature it is constantly associated to the interaction of genetic factors and lifestyle. Secondary hypertension emerges in earlier ages usually resulting from other factors as the diseases with effect on blood pressure values (e.g., kidney diseases)^23^.

In Brazil the prevalence of SAH among seniors is estimated at over 50.0%, with no significant changes in the five-year period^17^. In the city of São Paulo, Mendes et al.^18^ showed prevalence of SAH greater than 45.0% in the elderly population.

The prevalence of SAH is also high in the international context. In the United States about 30% of the total adult population is affected by SAH; for individuals aged 60 years or older, this percentage is 65%^20^. These values become even more concerning when considered that high blood pressure is the greatest factor contributing to the global burden of diseases and to mortality in the world^23^.

The development process of populations has been strongly associated to the increased prevalence of SAH^23^, since this clinical condition is connected to the increase in longevity, excessive salt intake, decrease in the practice of exercises and in consumption of fresh fruits and vegetables. For the next decade, the number of SAH patients worldwide is expected to increase significantly – reflecting not only the growth and aging of the world population, but also the increased exposure to risk factors, especially in developing countries^23^.

Increased number of individuals with SAH in populations has contributed to increase of treatments, both for nondrug treatments and for treatments based on use of antihypertensives^26^. However, nondrug treatment – when implemented isolatedly – is not always enough to lower blood pressure below the cut-off for diagnosis of SAH, considered as values greater than or equal to 140 millimeters of mercury (mmHg) of systolic pressure or 90 mmHg of diastolic pressure, according to the Brazilian Guidelines for Arterial Hypertension^26^.

In the pharmacological approach, despite conflicting recommendations as to the type of first-choice antihypertensive^23^, the drug treatment starts with the prescription of a class of antihypertensive advancing to the replacement between classes of medicines until the introduction of combined treatments of two or more types of antihypertensives, if no result is observed in the first interventions^26^. In this context, there are five classes of antihypertensives with proven effectiveness in the treatment of SAH and in the reduction of morbidity and mortality in hypertensive individuals: diuretics; beta blockers; angiotensin-converting enzyme (ACE) inhibitors; AT1 receptor blockers; and calcium channel blockers^26^.

Thus, knowledge of the factors associated with the use of antihypertensive among seniors can help in identifying more vulnerable groups, contributing to the planning of the distribution of antihypertensive drugs in Brazil and, consequently, to the treatment and control of arterial hypertension^26^.

Recent studies^3^ ^,^ ^16^ that evaluated the use of medications for the control of hypertension in Brazilian adults showed use of antihypertensives in level similar to that found in other countries. According to study by Chor et al.^3^, of the medicines used most belonged to the classes recommended in the national and international guidelines for hypertension, with greater use of diuretics. Almost 60.0% of participants made use of two or more antihypertensives to control blood pressure levels.

However, researches that associate the use of antihypertensives with socioeconomic and behavioral characteristics among Brazilian seniors are scarce, especially in relation to using a longitudinal approach. Thus, this study aimed to analyze the change in the use of antihypertensives over the last decade (2000 to 2010) among seniors. We also analyzed the association between the use of antihypertensives and socioeconomic and behavioral characteristics of the elderly population.

## METHODS

This seriate cross-sectional study is part of the *Saúde, Bem-Estar e Envelhecimento* study (SABE – Health, Well-being, and Aging), which collected data from a representative sample of seniors living in the city of São Paulo, in 2000, 2006, and 2010. The data were obtained using household interviews, by employing a questionnaire consisting of 11 thematic blocks covering information on personal data, cognitive evaluation, health conditions, functional status, anthropometric data, mobility test, use of medications, use and access to services, family and social support network, labor history, and housing characteristics^15^.

The study comprised a probabilistic sample, stratified by sex and age group, obtained by drawing households based on permanent record of 72 census tracts generated based on the *Pesquisa Nacional por Amostra de Domicílios* (PNAD – National Household Sample Survey). Conglomerate sampling method was used under the criterion of probability proportional to the number of households, in addition to oversample corresponding to the compensatory increase of mortality in the population aged over 75 years, with the objective of achieving the desired number of interviews for this age group^15^.

The dependent variable used in this analysis was the use of antihypertensives, obtained from the question “Could you show me the medicines that you are currently using or taking?” and, subsequently, from the names in the packaging of medicines and prescriptions – if there were any – shown by the respondent. The pharmaceutical form of medicines and the quantity consumed were also registered. The medicines were categorized according to classification adopted by the WHO, called Anatomical Therapeutic Chemical (ATC), which discriminates the medicines according to composition, mechanism of action, and main applicability^28^. The codes for antihypertensives were separated in the SABE study database and used in the analysis according to the use of antihypertensives, considering the classes with proven efficiency in the maintenance of blood pressure values: beta blockers; diuretics; calcium channel blockers; ACE inhibitors; angiotensin II receptor antagonists; and others (which include centrally acting adrenergic inhibitors, alpha-1 blockers, dilators, among others that result in lowering arterial blood pressure). Moreover, information about the use of antihypertensive was compared to the report of presence of SAH in the three periods, observing the association of this type of medicine for the specific treatment of arterial hypertension in the seniors of the samples.

We analyzed the following socioeconomic and demographic variables: sex; age in years (60-64; 65-69; 70-74; 75-79; 80-84; 85 years or older); *per capita* income (number of minimum wages in the year of data collection); skin color (obtained from the respondent’s self-declaration, according to predefined categories: white, brown, black, and others). Education level was informed according to the number of years of formal study, corresponding to the last grade in which the respondent obtained approval. For information on marital status and marital coexistence, two categories were used: no companion and with companion.

Behavioral characteristics analyzed were alcohol intake, smoking history, body mass index (BMI), and practice of physical activity. Data on alcohol intake were obtained from the recall of three months prior to the interview, using the dichotomous answer on consumption (yes; no). Smoking history was divided into three categories: never smoked, has smoked, and smokes currently. BMI was estimated by using the ratio of the individual’s weight over the height squared and categorized in low weight, adequate weight, and overweight, according to recommendation of the Food and Nutrition Surveillance Technical Standard for values referring to seniors^19^. Data on physical activity were obtained using the International Physical Activity Questionnaire (IPAQ), short version, validated instrument to collect data on the practice of physical activity^12^. Information on the practice of physical activity was categorized according to duration in minutes and intensity, using the criteria described by Hallal^12^, by establishing a cut-off point between physically active and insufficiently active of 150 minutes of minimum weekly practice, based on the guidelines for the practice of physical activity of the American College of Sports Medicine (ACSM)^22^.

For statistical analysis we used multilevel logistic regressions with the aim of controlling by the clustering of individuals, assuming that the observations are not completely independent (in this case, seniors aggregated in the different districts of the city). The model’s first level was composed by the individuals and the second by the districts of residence of the seniors. Odds ratios were estimated with statistical fit for the multiple factors entered into the model, following a conceptual framework described by Victora et al.^27^, where the variables in the multivariate model fit the data inserted later into the model. Thus, the hierarchy used starts in the sociodemographic characteristics, then following to the behavioral and economic characteristics. The three periods (2000, 2006, and 2010) were analyzed first separately and then together, aiming to analyze the change of the probability of use of antihypertensive by the inclusion of year dummies in the multilevel model.

Bayesian inference was used to estimate the model parameters due to the dichotomous nature of the variable *answer* (use of antihypertensive), an approach recommended to decrease the bias inherent in the use of maximum-likelihood procedures in multilevel analyses^25^. The analysis also enables the comparison of quality of fit of the models by calculating the Bayesian information criterion (BIC) coefficient, in which lower values indicate better fit of the model in relation to the variable *answer* ^25^.

Analyses were performed on the Stata 13.1 program (Stata Corp., College Station, Texas, 2013) using the *melogit* command for multilevel analysis of mixed effects.

This study was approved by the Research Ethics Committee of the *Faculdade de Saúde Pública* at the *Universidade de São Paulo* (Process 67/1999) and by the National Committee of Ethics in Research (CONEP – Process 315/1999).

## RESULTS

Samples per segment were composed of 2,143, 1,413, and 1,344 individuals of both sexes, aged 60 years or older, residing in the city of São Paulo, for the samples of 2000, 2006, and 2010, respectively.

Of the total respondents, for the three periods, most were female (59.0% in 2000, 61.8% in 2006, and 64.2% in 2010). The classification as for living with or with no partner indicated relatively equivalent division between the two categories. The report of white skin color was the most frequent in the three years. Over the period, there was constant decrease in the proportion of seniors with no year of education level. Most seniors had income of less than two minimum wages in the three waves (Table).

**Table 1:** Distribution of seniors according to demographic, socioeconomic, and behavioral characteristics in the segments 2000, 2006, and 2010. São Paulo, SP, Southeastern Brazil.

Variable	2000 (n = 2,143)*		2006 (n = 1,413)*		2010 (n = 1,344)*	
		
	n	%	n	%	n	%
Sex						
Female	878	41.0	540	38.2	481	35.8
Male	1,265	59.0	873	61.8	863	64.2
Age (years)						
60-64	426	19.9	298	21.1	389	30.6
65-69	379	17.7	237	16.8	194	15.3
70-74	336	15.7	208	14.7	213	16.8
75-79	472	22.0	232	16.4	148	11.6
80-84	307	14.3	230	16.3	150	11.8
≥ 85	223	10.4	208	14.7	178	14.0
Skin color						
White	1,524	71.5	930	65.9	781	58.7
Brown	246	11.5	258	18.3	387	29.1
Black	86	4.0	101	7.2	94	7.1
Others	275	12.9	122	8.6	69	5.2
Education level (years)						
0	502	23.6	268	19.0	205	15.3
1-3	572	26.8	392	27.8	326	24.3
4-7	730	34.3	514	36.5	496	36.9
≥ 8	327	15.3	235	16.7	316	23.5
Current marital status						
With companion	1,122	52.4	694	48.2	664	50.0
No companion	1,020	47.6	717	50.8	664	50.0
Income (in minimum wages)						
< 2	1,115	54.6	709	50.2	782	66.5
≥ 2	928	45.4	704	49.8	394	33.5
Alcohol intake in the last three months						
No	1,514	70.6	1,046	74.2	959	71.4
Yes	629	29.4	363	25.8	384	28.6
Smoking						
Never smoked	1,155	53.9	770	54.5	707	52.6
Has smoked	697	32.6	486	34.4	493	36.7
Smoking currently	290	13.5	157	11.1	143	10.7
BMI						
Low weight	312	17.4	231	17.4	126	10.4
Adequate weight	770	42.9	555	41.9	431	35.6
Overweight	714	39.7	537	40.6	653	54.0
Practice of recommended PA (150 min/week)						
No	1,655	77.3	603	42.7	822	61.2
Yes	487	22.7	810	57.3	522	38.8

BMI: body mass index; PA: physical activity

* Total number of individuals in the sample.

As to behavioral characteristics, about 70.0% of individuals reported no alcohol intake in the previous three months in the three periods analyzed. As to use of tobacco, slightly over half of the seniors reported never having smoked and a small proportion of seniors (10.7% to 13.5%) reported smoking currently. According to the BMI we observed increased prevalence of overweight seniors from 2000 to 2010. As to prevalence of recommended practice of physical activity (at least 150 minutes a week), except for the sample of 2006, most seniors were considered insufficiently active (Table).

Distribution of the population according to use of antihypertensives indicates increasing prevalence over the three periods (Table 2). The same increase trend was observed in the adoption of combined drug treatment, totaling more than 90.0% in 2010. More than 80.0% of seniors who reported being hypertensive made use of medications for lowering arterial blood pressure in the three periods (Table 2).

**Table 2:** Distribution of seniors according to use of antihypertensive and combined therapy in the segments 2000, 2006, and 2010. São Paulo, SP, Southeastern Brazil.

Variable	2000 (n = 2,143)*		2006 (n = 1,413)*		2010 (n = 1,344)*	

	n	%	n	%	n	%
Use of antihypertensive						
No	1,099	51.3	547	38.7	461	34.3
Yes	1,044	48.7	866	61.3	883	65.7
Use of combined therapy						
No	314	31.1	151	17.4	74	8.4
Yes	730	69.9	715	82.6	809	91.6
Use of antihypertensive among hypertensive seniors						
No	219	19.2	116	12.8	77	8.5
Yes	924	80.4	788	87.2	830	91.5

* Total number of individuals in the sample.

Table 3 presents the multilevel regression analysis for use of antihypertensives, conducted separately for 2000, 2006, and 2010, which showed similar results for the three years. As to sociodemographic characteristics, we observed consistent and statistically significant association between use of antihypertensives and sex, age, black and brown skin color, and living with no companion. In the three periods, use of antihypertensives was significantly higher among females. We observed increased use of antihypertensives in ages over 60-64 years. As to skin color, use of antihypertensives was significantly higher among individuals who reported black skin color compared with those who reported white skin color in the three periods. Reported brown skin color showed significant association with higher use of antihypertensives in 2006. As for marital coexistence, seniors who reported having no companion had less chance of using drugs to reduce arterial blood pressure in 2010. As to socioeconomic variables, specifically education level and income, we observed no statistically significant association with use of antihypertensives in the three periods under analysis.

**Table 3:** Multilevel logistic regression models for use of antihypertensives according to demographic, socioeconomic, and behavioral characteristics in seniors, analyzed separately for 2000, 2006, and 2010. São Paulo, SP, Southeastern Brazil.

Variable	2000		2006		2010	
			
1st Level	OR	95%CI	OR	95%CI	OR	95%CI
Sex						
Female	1.72^b^	1.39–2.13	1.57^b^	1.23–2.02	1.57^a^	1.14–2.15
Age (years)						
65-69	1.55^a^	1.16–2.08	1.24	0.87–1.77	1.54^a^	1.03–2.31
70-74	1.37^a^	1.01–1.85	2.72^b^	1.84–4.02	1.97^b^	1.32–2.94
75-79	1.69^b^	1.27–2.25	2.14^b^	1.48–3.11	2.86^b^	1.77–4.63
80-84	1.78^b^	1.29–2.45	2.25^b^	1.54–3.28	3.47^b^	2.12–5.68
≥ 85	1.53^a^	1.07–2.20	2.29^b^	1.54–3.42	2.45^b^	1.55–3.88
Skin color						
Brown	1.28	0.96–1.70	1.44^a^	1.06–1.96	1.14	0.84–1.56
Black	1.69^a^	1.07–2.67	2.28^b^	1.41–3.68	1.77^a^	1.01–3.11
Others	0.90	0.69–1.18	0.97	0.65–1.44	0.71	0.40–1.27
Education level						
1-3 years	1.08	0.84–1.39	1.29	0.93–1.81	0.77	0.50–1.19
4-7 years	0.95	0.74–1.22	1.32	0.95–1.83	0.95	0.62–1.46
≥ 8	0.91	0.66–1.26	1.18	0.79–1.76	0.83	0.51–1.37
Current marital status						
No companion	0.83	0.68–1.02	0.78	0.60–1.01	0.70^a^	0.51–0.95
Income (in minimum wages)						
≥ 2	1.01	0.82–1.23	1.14	0.90–1.46	0.89	0.65–1.22
Alcohol intake in the last three months						
Yes	0.71^a^	0.56–0.90	0.82	0.61–1.09	0.71^a^	0.52–0.97
Smoking						
Has smoked	1.40^a^	1.08–1.80	1.52^a^	1.13–2.04	1.41^a^	1.07–2.03
Smoking currently	0.78	0.56–1.09	0.68	0.45 –1.02	0.74	0.47–1.16
BMI						
Adequate weight	1.37^a^	1.02–1.83	1.29	0.93–1.81	1.06	0.67–1.69
Overweight	2.76^b^	2.03–3.76	2.34^b^	1.64–3.33	2.16^b^	1.35–3.44
Practice of recommended PA (150 min/week)						
Yes	0.85	0.67–1.09	0.59^b^	0.46–0.78	0.82	0.62–1.10

**2nd Level Districts of residence**						

BIC	2,383.63		1,760.57		1,375.13	

BMI: body mass index; PA: physical activity; BIC: Bayesian information criterion

^a^ p < 0.05.

^b^ p ≤ 0.001.

In the analysis of behavioral variables (Table 3), alcohol intake and smoking showed significant association with use of medicines. Alcohol intake was associated with lower use of antihypertensives in 2000 and 2010. As to smoking, having smoked was associated with higher use of medicine. On the other hand, seniors who reported smoking currently showed no association with use of antihypertensive. As to BMI, values considered as overweight showed significant association with higher use of antihypertensives. As to practice of physical activity, analysis of the three periods pointed to statistically significant association with use of antihypertensives only in 2006.

Finally, aggregate analysis was carried out with inclusion of results of the three waves to analyze if there was statistically significant difference for use of antihypertensive in 2000, 2006, and 2010 (Table 4). Model 2 indicates that, after controlling for socioeconomic and behavioral characteristics, the likelihood of using antihypertensives increased both for 2006 and in 2010, in relation to 2000. For 2006, we observed an odds ratio 1.90 (95%CI 1.60–2.24) times higher of using antihypertensive in relation to 2000. For 2010 odds ratio was even higher (OR = 1.94; 95%CI 1.62–2.33).

**Table 4:** Multilevel logistic models for use of antihypertensive according to demographic, socioeconomic, behavioral characteristics among seniors, aggregate analysis for 2000, 2006, and 2010. São Paulo, SP, Southeastern Brazil.

Variable	Empty Model (n = 4,899)		Model 1 (n = 4,899)		Model 2 (n = 4,899)	
			
1st Level	OR	95%CI	OR	95%CI	OR	95%CI
Intercept	1.33^b^	1.23–1.43	0.34^b^	0.24–0.48	0.28^b^	0.20–0.41
Sex						
Female			1.56^b^	1.31–1.86	1.60^b^	1.35–1.92
Age (years)						
65-69			1.43^b^	1.16–1.78	1.43^b^	1.15–1.77
70-74			1.85^b^	1.48–2.31	1.80^b^	1.44–2.25
75-79			1.97^b^	1.57–2.47	1.97^b^	1.58–2.47
80-84			2.40^b^	1.87–3.06	2.24^b^	1.75–2.87
≥ 85			2.51^b^	1.91–3.31	2.17^b^	1.65–2.86
Skin color						
Brown			1.36^b^	1.13–1.64	1.22^a^	1.01–1.47
Black			2.02^b^	1.49–2.73	1.85^b^	1.36–2.51
Others			0.81	0.65–1.02	0.88	0.70–1.10
Education level (years)						
1-3			1.15	0.94–1.40	1.09	0.89–1.33
4-7			1.22^a^	1.01–1.49	1.11	0.91–1.36
≥ 8			1.14	0.89–1.46	0.97	0.75–1.25
Current marital status						
No companion			0.83^a^	0.72–0.97	0.81^a^	0.70–0.95
Income (in minimum wages)						
≥ 2			0.93	0.80–1.08	0.97	0.83–1.13
Alcohol intake in the last three months						
Yes			0.70^b^	0.60–0.82	0.72^b^	0.62–0.84
Smoking						
Has smoked			1.54^b^	1.31–1.82	1.51^b^	1.27–1.78
Smokes currently			0.75^b^	0.60–0.94	0.75^a^	0.60–0.94
BMI						
Adequate weight			1.34^a^	1.10–1.63	1.32^a^	1.08–1.60
Overweight			2.69^b^	2.19–3.30	2.57^b^	2.09–3.16
Practice of recommended PA (150 min/week)						
Yes			0.89	0.77–1.02	0.74^b^	0.63–0.86
Year						
2006					1.90^b^	1.60–2.24
2010					1.94^b^	1.62–2.33

BIC	6,708.26		5,313.15		5,252.98	

BMI: body mass index; PA: physical activity; BIC: Bayesian information criterion

^a^ p < 0.05.

^b^ p ≤ 0.001.

## DISCUSSION

This study enabled the analysis of use of antihypertensives and its association with sociodemographic, economic, and behavioral variables among the elderly. Female sex, old age, black skin color, alcohol intake, smoking history, and nutritional evaluation of overweight were the variables most consistently associated with use of antihypertensives. The study also identified that, even after controlling for sociodemographic and behavioral variables, the use of antihypertensive drugs has increased significantly among the elderly of the city of São Paulo over the last decade (2000 to 2010).

Women had higher probability of use of antihypertensive drugs in relation to men, which can be explained by the higher attention and use of health care related to women^21^.

The association between old age and higher use of antihypertensives observed in this study may be related with the appearance of problems and diseases related with health, as well as the increased risk of developing SAH related with the advancement of age^8^.

Regarding skin color, we observed association between reported black skin color and higher use of antihypertensives. Such finding can be justified based on the higher prevalence of SAH acknowledgedly associated with black populations and their descendants^23^, although Agyemang et al.^1^ have reported that genetic characteristics linked to ethnicity may not act as determinants, but as factors predisposing to the development of SAH. However, the relation between the higher use of antihypertensives among black seniors in Brazil has not been analyzed in the literature. This information has important relevance in public health, since both the access to health services and the distribution of income in this portion of the population can influence the purchase of medicines, the access to adequate treatment, and the inequalities in health.

Use of alcohol is considered a risk factor for SAH^26^. However, alcohol intake, especially in excess, often has inverse relation with the care of health^6^ and with lower adherence to drug treatment of SAH^10^. In this context, the statistically lower use of antihypertensives among seniors who reported drinking alcohol observed in this study may help identify a risk group of the elderly population, assisting in the planning of strategies and policies of prevention and health care for the elderly.

Smoking, similarly to SAH, is among the most important risk factors for cardiovascular diseases^26^. However, the relation between smoking and hypertension is the subject of controversial results in the literature. Recently, D’elia et al.^7^, in a longitudinal study, described the lower risk of developing SAH among individuals who have never smoked and former smokers compared with smokers. In this study, we observed a consistent association between smoking history and higher use of antihypertensives, indicating that having quit smoking may be related to increased concern and care in relation to the health status, as with the use of medicines.

As to nutritional status, we found association between overweight and higher use of antihypertensives in the three periods analyzed (2000, 2006, and 2010). However, in analyzing the interaction between BMI and presence of SAH, we found no significant differences for use of antihypertensives between hypertensive individuals with overweight and with adequate weight. In this context an important aspect to be considered is the high use of antihypertensives for efficient control of blood pressure values, since the drug control of hypertension in obese individuals is more difficult, requiring higher use of antihypertensives^4^.

Previous studies have pointed to an inverse association between practice of physical activity and prevalence of high blood pressure values^5^. However, the results found in this study were not conclusive in this regard. We found significant association only in the 2006 wave, in which the recommended practice of physical activity was associated with lower use of antihypertensive drugs.

Use of more than one type of antihypertensive to control SAH was common among the seniors, a result similar to that found in investigation in the elderly population in Minas Gerais^11^. Considering that over 80% of the hypertensive individuals evaluated use antihypertensives, the results of this study are an important aspect to be considered in the discussion on treatment methods for SAH and on the use of medications in the elderly, since the concomitant use of various types of medicines can cause harmful interactions and accumulation of adverse effects^9^.

This study also showed statistically significant increase for use of antihypertensives among seniors over the last decade (2000 to 2010), even after controlling for changes in the sociodemographic and behavioral variables. Due to accumulation of diseases in seniors, increased use of medicines will be a major challenge for the health system, since, with populational aging and the proportional growth of chronic diseases, medications are an important tool for treatment, recovery and prevention^2^.

This study presents limitations to be considered. Data from questionnaires answered by seniors should always be used with caution due to the possible presence of memory bias. Additionally, although the collection instrument for physical activity (IPAQ short version) has been validated, there may be problems of reproducibility of results^13^. Another limitation is the absence of an instrument to assess adherence to medication – as the test of Morisky-Green – in the questionnaire of the SABE study, which made the analysis of adherence to antihypertensive treatment not viable.

The results of this study highlight the importance of attention as to the use of medications in the elderly and to the treatment of chronic diseases of high prevalence in this population. Analysis of the results for the largest city in Latin America may reflect the future situation of seniors in the region and the need for planning and adjustment of health care based on the effectiveness of care and on the efficiency of the expenditure on public health.

Decrease in the use of antihypertensives by changing behaviors that are harmful to health – such as excessive alcohol intake, smoking, and overweight – may contribute to improve the epidemiological context of chronic diseases and avoid drug interactions, improving the quality of life of the Brazilian elderly population.
